# Induction of sodium iodide symporter gene and molecular characterisation of HNF3*β*/FoxA2, TTF-1 and C/EBP*β* in thyroid carcinoma cells

**DOI:** 10.1038/sj.bjc.6604544

**Published:** 2008-08-05

**Authors:** T Akagi, Q T Luong, D Gui, J Said, J Selektar, A Yung, C M Bunce, G D Braunstein, H P Koeffler

**Affiliations:** 1 Division of Hematology and Oncology, Department of Medicine, Cedars-Sinai Medical Center, UCLA School of Medicine, Los Angeles, CA, USA; 2 Samuel Oschin Comprehensive Cancer Center, Cedars-Sinai Medical Center, UCLA School of Medicine, Los Angeles, CA, USA; 3 Department of Pathology, UCLA School of Medicine, Los Angeles, CA, USA; 4 School of Biosciences, University of Birmingham, Birmingham, B15 2TT, UK; 5 Division of Endocrinology, Department of Medicine, Cedars-Sinai Medical Center, UCLA School of Medicine, Los Angeles, CA, USA

**Keywords:** thyroid cancer, papillary, anaplastic, HNF3*β*/FoxA2, C/EBP*β*

## Abstract

Thyroid carcinoma cells often do not express thyroid-specific genes including sodium iodide symporter (NIS), thyroperoxidase (TPO), thyroglobulin (TG), and thyrotropin-stimulating hormone receptor (TSHR). Treatment of thyroid carcinoma cells (four papillary and two anaplastic cell lines) with histone deacetylase inhibitors (SAHA or VPA) modestly induced the expression of the NIS gene. The promoter regions of the thyroid-specific genes contained binding sites for hepatocyte nuclear factor 3 *β* (HNF3*β*)/forkhead box A2 (FoxA2), thyroid transcription factor 1 (TTF-1), and CCAAT/enhancer binding protein *β* (C/EBP*β*). Quantitative reverse transcription-polymerase chain reaction (RT–PCR) showed decreased expression of HNF3*β*/FoxA2 and TTF-1 mRNA in papillary thyroid carcinoma cell lines, when compared with normal thyroid cells. Forced expression of these genes in papillary thyroid carcinoma cells inhibited their growth. Furthermore, the CpG island in the promoter region of *HNF3β/FoxA2* was aberrantly methylated; and treatment with 5-aza-2-deoxycytidine (5-Az) induced its expression. Immunohistochemical staining showed that C/EBP*β* was localised in the nucleus in normal thyroid cells but was detected in the cytoplasm in papillary thyroid carcinoma cells. Subcellular fractionation of papillary thyroid carcinoma cell lines also demonstrated high levels of expression of C/EBP*β* in the cytoplasm, suggesting that a large proportion of C/EBP*β* protein is inappropriately localised in the cytoplasm. In summary, these findings reveal novel abnormalities in thyroid carcinoma cells

Development of thyroid carcinoma is accompanied by a block of differentiation of these cells. Papillary and follicular thyroid carcinomas initially are relatively well-differentiated tumours that over time may de-differentiate, whereas anaplastic thyroid carcinoma is an undifferentiated tumour (Farid *et al*, 1994). Poorly differentiated or undifferentiated carcinomas no longer express mature thyroid-specific genes including sodium iodide symporter (NIS), thyroperoxidase (TPO), thyroglobulin (TG), and thyrotropin-stimulating hormone receptor (TSHR). In normal thyroid cells, TSHR is stimulated by thyrotropin-stimulating hormone, resulting in the activation of NIS, which incorporates iodine. Thyroglobulin and iodine are catalysed into thyroid hormones by TPO (Carrasco, 1993). Investigators have predominantely attempted to induce differentiation of thyroid cancer cells by exposure to compounds associated with known differentiation of other cancers.

Retinoic acid, including *all-trans* retinoic acid (ATRA) and *9-cis* retinoic acid (9-cis RA), induces differentiation of acute promyelocytic leukaemia cells and neuroblastoma cells and is used in the therapy for these cancers. Retinoids have been reported to induce the expression of TPO, TG, and NIS mRNAs in thyroid carcinoma cell lines (Schmutzler *et al*, 1997; Haugen, 2004). The *NIS* promoter contains CpG islands, and a DNA demethylating agent (such as, 5-aza-2-deoxycytidine (5-Az)) combined with a histone deacetylase inhibitor has been shown to induce NIS expression and radioactive iodine uptake in follicular and anaplastic thyroid carcinoma cell lines (Venkataraman *et al*, 1999; Haugen, 2004).

The transcription factors of paired box gene 8 (Pax-8) and thyroid transcription factor 1 (TTF-1) have been analysed in thyroid cells. Paired box gene 8 is necessary for the formation of thyroxine-producing follicular cells in the thyroid gland (Mansouri *et al*, 1998, 1999); and fusion of the *Pax-8* and peroxisome proliferator-activated receptor *γ* (*PPARγ*) genes occurs in approximately 30% of follicular thyroid carcinomas (Kroll *et al*, 2000; Dwight *et al*, 2003). Thyroid transcription factor 1 is required for the development of the thyroid gland, and *TTF-1*-deficient mice lack a thyroid gland and die at birth (Kimura *et al*, 1996). The expression of Pax-8 and TTF-1 is low in thyroid carcinoma (Ros *et al*, 1999); and stable transfection with a Pax-8 expression vector in anaplastic thyroid carcinoma cell line, ARO, caused re-expression of endogenous NIS, TG, and TPO (Presta *et al*, 2005). Reporter gene analysis found that the promoter region of *TG*, *TPO*, and *TSHR* could be activated by the forced expression of either Pax-8 or TTF-1 in the papillary thyroid carcinoma cell line NPA (Ros *et al*, 1999). Co-transfection of Pax-8 and TTF-1 restored *TG* promoter activity in WRO (follicular thyroid carcinoma) and ARO cells (Chun *et al*, 1998).

In this study, we attempted to induce differentiation of papillary and anaplastic thyroid carcinoma cells as measured by the induction of NIS, TPO, TG, and TSHR by exposing the cells to 5-Az, histone deacetylase inhibitors (suberoylanilide hydroxamic acid (SAHA) and valproic acid), ATRA, 9-cis RA, troglitazone (PPAR*γ* ligand), 1,25-dihydroxyvitamin D3 (1,25(OH)_2_D_3_), thyroid hormone T_3_, and thyrotropin-stimulating hormone. Furthermore, dysregulation of three transcription factors (TTF-1, hepatocyte nuclear factor 3 *β* (HNF3*β*)/forkhead box A2 (FoxA2), and CCAAT/enhancer binding protein *β* (C/EBP*β*)) was examined in thyroid carcinoma cells.

## Materials and methods

### Cell culture and drug treatments

BHP (sublines 2–7, 7–13, 10-3 and 18–21) and NPA papillary, and ARO and FRO anaplastic thyroid carcinoma cell lines were cultured as described before (Fagin *et al*, 1996; Ohta *et al*, 1997; Luong *et al*, 2006). The normal rat thyroid cell line FRTL-5 cells (from Dr Shlomo Melmed at Cedars-Sinai Medical Center) were cultured in Ham's F12K medium (Invitrogen, Carlsbad, CA, USA) with 5% bovine calf serum (Invitrogen) together with 10 mU ml^−1^ thyrotropin-stimulating hormone, 0.01 mg ml^−1^ insulin, 10 nM hydrocortisone, 5 ng ml^−1^ transferrin, 10 ng ml^−1^ somatostatin, and 10 ng ml^−1^ glycyl-L-histidyl-L-lysine acetate.

Cultured cells were treated with the following agents either alone or in combinations with 5-Az (1 *μ*M), SAHA (5 *μ*M), valproic acid (1 *μ*M), ATRA (100 nM), *9-cis* RA (100 nM), troglitazone (PPAR*γ* ligand, 10 *μ*M), 1,25(OH)_2_D_3_ (1 *μ*M), thyroid hormone T_3_ (10 nM), and thyrotropin-stimulating hormone (1 mU ml^−1^) for 48–96 h. 5-Aza-2-deoxycytidine, valproic acid, thyroid hormone T_3_, and thyrotropin-stimulating hormone were dissolved in water; SAHA and troglitazone were diluted in dimethyl sulphoxide (DMSO); ATRA, 9-cis RA, and 1,25(OH)_2_D_3_ were placed in ethanol. Equal volume of DMSO and ethanol were added in control samples.

### Real-time reverse transcription polymerase chain reaction

Total RNA was isolated from thyroid carcinoma cell lines and normal thyroid tissues using Trizol reagent (Invitrogen), and cDNA was prepared from 1 *μ*g of total RNA with Superscript III reverse transcriptase (Invitrogen). Expression of mRNAs was measured by real-time PCR using an iCycler iQ system (Bio-Rad, Hercules, CA, USA) as described previously (Xie *et al*, 2001). To determine the expression levels of NIS, C/EBP*α*, and C/EPB*β* with probes, amplification reactions were performed with the Universal Taqman PCR mastermix (Applied Biosystems, Foster City, CA, USA). Expression levels of TPO, TG, TSHR, HNF3*β*/FoxA2, TTF-1, and Pax-8 were measured with platinum Taq DNA Polymerase (Invitrogen) and SYBRGreen I (Molecular Probes, Carlsbad, CA, USA). Expression levels of target genes were normalised with 18S or *β*-actin. Specificity of all PCR products was checked on agarose gel, and only one product of the correct size was observed for each primer pair. Sequences of primers and probes, the melting temperature, and product size are shown in Table 1.

### Radioactive iodine (^125^I) uptake assay

Na^125^I (100 *μ*Ci *μ*l^−1^) stock was diluted to 0.02 *μ*Ci *μ*l^−1^ using Hanks' Balanced Salt Solution (HBSS). Cells were plated at 1 × 10^5^ cells per well in 12-well plates and treated with appropriate drugs, either with or without 10 mU ml^−1^ thyrotropin-stimulating hormone and/or cold (unlabelled) NaI. For radioactive iodine uptake, cells were washed twice with HBSS and 500 *μ*l of Na^125^I working solution was added. After incubation for 3 h at 37°C, cells were washed with 1 ml cold HBSS and lysed with 1 ml of 95% ethanol for 1 h at 37°C. Lysates were transferred to vials for counting, and total counts were normalised to number of viable cells in parallel cultures.

### Plasmid transfection and colony assay

Expression vectors of pTTF-1 and pHNF3*β*/FoxA2 were generous gifts from Dr Edward Morrisey (University of Pennsylvania). Plasmids of 1 *μ*g pTag1 (empty vector), pTTF-1, and pHNF3*β*/FoxA2 were transfected into BHPs cells using Lipofectamine 2000 (Invitrogen). The zinc-inducible pMT-C/EBP*β* expression plasmid (Gery *et al*, 2005) was transfected into BHP cells (sublines 2–7 and 7–13), which were selected with 500 *μ*g ml^−1^ G418 for 48 h.

For colony assay, cells transfected with plasmid were plated at 1 × 10^5^ cells per well in 12-well plates in 1 ml culture media containing 500 *μ*g ml^−1^ G418. After 2 weeks, cells were stained with Crystal violet dye (0.25% crystal violet dissolved in 50% methanol).

### 3-(4,5-Dimethylthiazol-2-yl)-2,5-diphenyltetrazolium bromide and clonogenic soft agar assay

Cells were treated with 10 *μ*l of 5 mg ml^−1^ MTT (3-(4,5-dimethylthiazol-2-yl)-2,5-diphenyltetrazolium bromide; Sigma-Aldrich, St Louis, MO, USA), and incubated at 37°C for 4 h. Medium was removed, and 50 *μ*l DMSO was added to the cells to solubilise the MTT. Plates were read at wavelength of 540 nm on a plate reader.

For clonogenic soft agar assays, cells were plated into 24-well flat-bottomed wells using a two-layer soft agar system with 1 × 10^3^ cells per well in a volume of 400 *μ*l per well as previously described (Luong *et al*, 2006). After 14 days of incubation, colonies were counted.

### Methylation analysis of *HNF3*β*/FoxA2* gene

Genomic DNA was modified by sodium bisulphate using EZ DNA Methylation Kit (Zymo Research, Orange, CA, USA). The CpG island (−761 to −561, ATG codon considered as +1) of the *HNF3*β*/FoxA2* gene was amplified from the bisulphate-modified genomic DNA with specific primers (sense primer: 5′-TTTTAGGGGATTTGTTGTGG-3′, anti-sense primer: 5′-AAATAATCAACTCACACC-3′). For PCR amplification, a total volume of 10 *μ*l was used containing modified genomic DNA, 0.5 *μ*M of each primers, 5.0 *μ*l of FailSafe PCR 2 × PreMixe E (Epicentre Biotechnologies, Madison, WI, USA) and 1.0 U platinum Taq (Invitrogen). Polymerase chain reaction products were subcloned into pCR 2.1 vector (Invitrogen) and sequenced.

### Subcellular fractionation, Western blot analysis, and immunohistochemistry

Total cell lysates were prepared by lysing cells in RIPA buffer (1% Nonidet P-40, 0.5% sodium deoxycholate, 0.1% SDS, 50 mM Tris-HCl (pH 7.5)) containing a protease inhibitor cocktail (Roche Diagnostics GmbH, Mannheim, Germany) as well as 1 mM NaF and 1 mM NaVO_4_. To separate nuclear and cytoplasmic fractions, cells were fractionated with NE-PER Nuclear and Cytoplasmic Extraction Reagent (Pierce Biotechnology, Rockford, IL, USA). These samples were subjected to sodium dodecyl sulphate–polyacrylamide gel electrophoresis (SDS–PAGE) followed by an electrotransfer to polyvinylidene difluoride membrane. The signals were developed with either Supersignal West Pico Chemiluminescent or Supersignal West Dura Extended Duration Substrate (Pierce Biotechnology). Anti-PPAR*γ*, C/EBP*β*, *β*-actin, and heterogeneous nuclear ribonuclear protein (hnRNP) A1 antibodies were obtained from Santa Cruz Technology (Santa Cruz, CA, USA). Anti-glyceraldehyde-3-phosphate dehydrogenase (GAPDH) was from Research Diagnostics Inc. (Concord, MA, USA).

Normal and papillary thyroid carcinoma tissue blocks were cut at 3 *μ*M thickness, deparaffinised and pre-treated in Tris-HCl (pH 9.0). These samples were incubated overnight with anti-C/EBP*β* antibody (1 : 500 dilution), washed, followed by the HRP-conjugated secondary antibody (Dako, Carpinteria, CA, USA), and DAB chromogen. The tissues were counterstained with haematoxylin and then coverslipped. Three samples of normal and three carcinomas were examined.

## Results

### Induction of expression of thyroid-specific genes: effect of 5-Az, SAHA, valproic acid, and nuclear hormone receptor ligands in thyroid carcinoma cell lines

Silencing of genes can be associated with epigenetic change including abnormal methylation of CpG islands and/or deacetylation of histones. Therefore, papillary (BHP sublines 2–7, 7–13, 10-3, and 18–21) and two anaplastic thyroid carcinoma (ARO and FRO) cell lines were cultured either with or without 5-Az (1 *μ*M) and/or histone deacetylase inhibitors (SAHA (5 *μ*M) or valproic acid (1 *μ*M)). Quantitative reverse transcription-polymerase chain reaction (RT–PCR) showed that expressions of thyroid-specific genes (TPO, TG, and TSHR) were either extremely low or at undetectable levels compared with normal thyroid tissue (data not shown).

**Figure 1:**
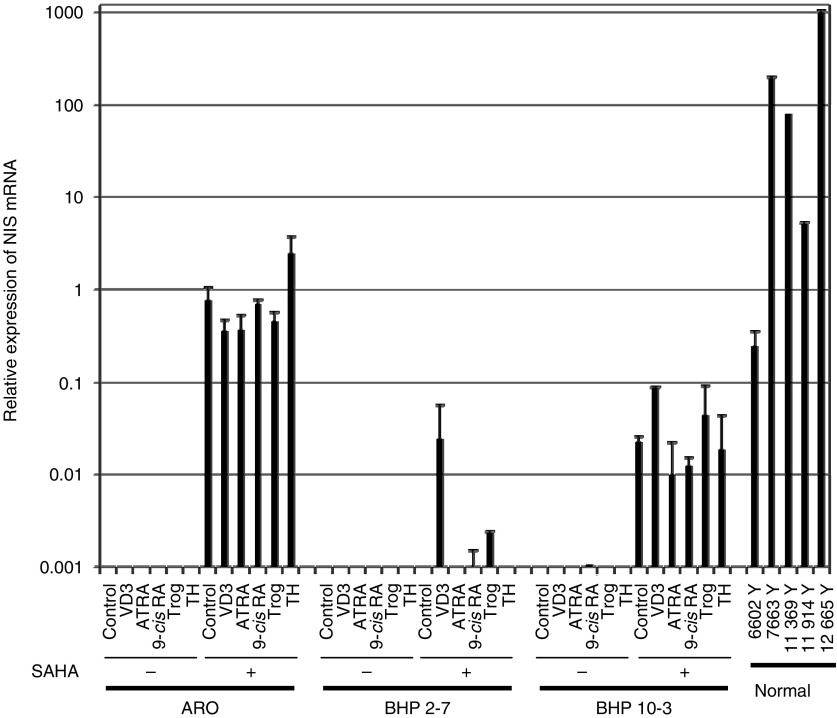
Induction of NIS expression in anaplastic and papillary thyroid carcinoma cell lines with SAHA and ligands of nuclear hormone receptors. Thyroid carcinoma cells (ARO and BHP) were cultured with or without 1,25(OH)_2_D_3_ (VD3, 1 *μ*M), *all-trans* retinoic acid (ATRA, 100 nM), *9-cis* retinoic acid (9-cis RA, 100 nM), troglitazone (Trog, 10 *μ*M), or thyroid hormone T_3_ (TH, 10 nM), either with or without SAHA (5 *μ*M). Expression of NIS mRNA in these cells was measured by quantitative RT–PCR after 48 h exposure. NIS expression was compared with levels present in normal thyroid cells. Relative levels of transcripts for NIS were normalised to 18S RNA transcripts within each sample. Level of NIS expression is the mean of *n*=3 samples.

We also examined the effects of several ligands of nuclear hormone receptors either with or without SAHA. As shown in Figure 1, 1,25(OH)_2_D_3_, ATRA, 9-cis RA, troglitazone, or thyroid hormone T_3_ alone was able to induce the expression of NIS mRNA in ARO and BHP (sublines 2–7 or 10–3) cells. The combination of SAHA with ATRA, 9-cis RA, troglitazone, or thyroid hormone T_3_ did not enhance the expression of NIS compared with SAHA alone in BHP papillary thyroid carcinoma cells (sublines 2–7 and 10–3).

### Thyroid-related transcription factors are poorly expressed in papillary and anaplastic thyroid carcinoma cell lines

**Figure 2:**
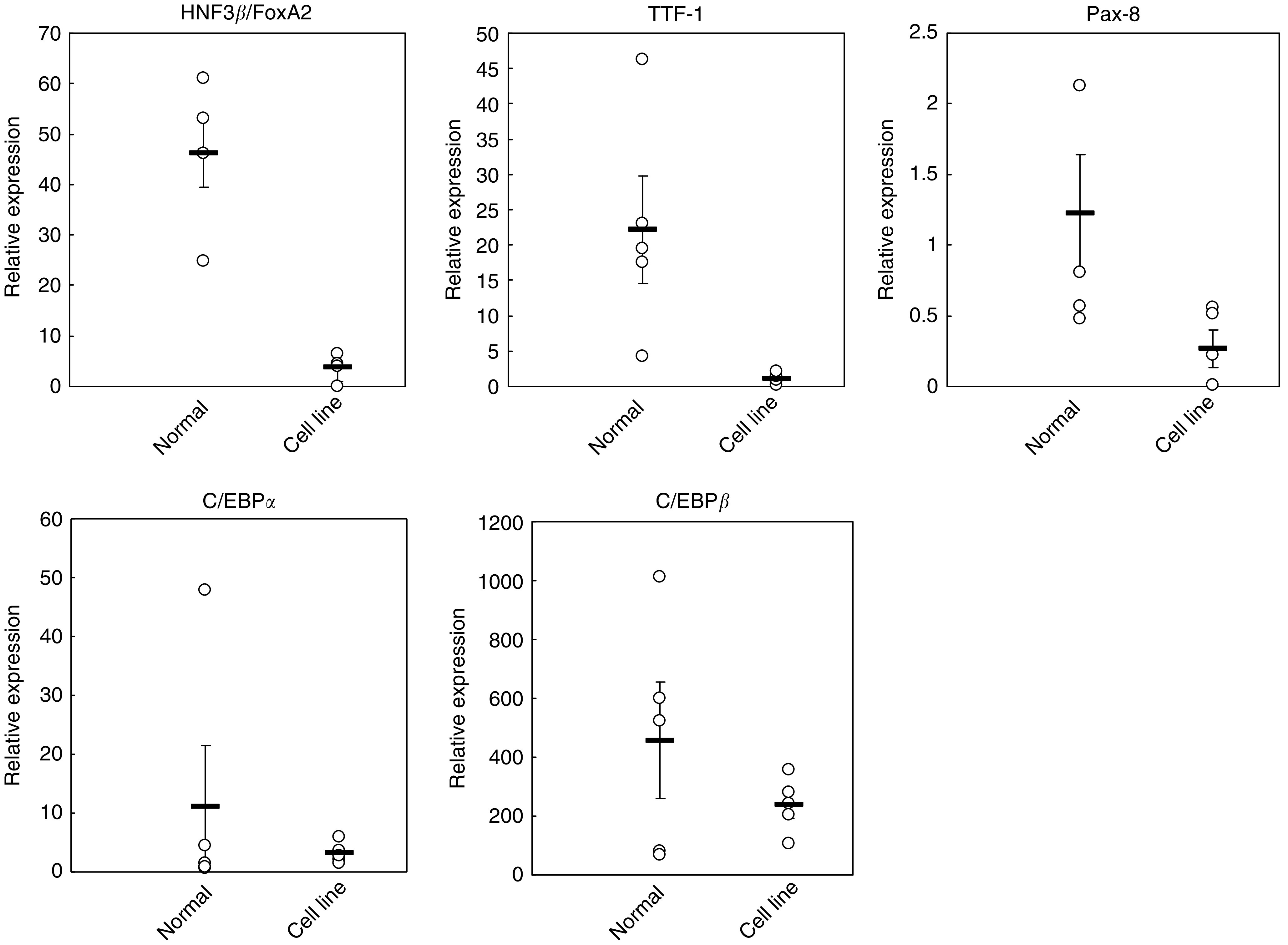
Expression of transcription factors in normal thyroid cells and thyroid carcinoma cell lines. Expression of transcription factors including HNF3*β*/FoxA2, TTF-1, Pax-8, C/EBP*α*, and C/EBP*β* was measured by quantitative RT–PCR in five normal thyroid samples and five thyroid carcinoma cell lines (BHPs 2–7, 7–13, 18–21, ARO, and FRO). Expression in individual samples or cell lines is represented by open circles, mean expression of normal or cancer cells is represented by the horizontal bar±s.e.

The above data showed that the four thyroid-specific genes (NIS, TPO, TG, and TSHR) were negligibly expressed in the thyroid carcinoma cell lines; therefore, common transcription factor(s) that regulate these genes might not be expressed in these cell lines. The promoter regions of these genes contain putative transcription factor binding sites for HNF3*β*/FoxA2, C/EBPs, PPAR*γ*, Pax-8, and TTF-1. Real-time PCR showed that HNF3*β*/FoxA2, TTF-1, and Pax-8 were expressed in normal thyroid tissue; in contrast, levels were either very low or undetectable in thyroid carcinoma cell lines (Figure 2). Western blot analysis showed that the protein expression of PPAR*γ* was barely detectable in BHP sublines, but easily found in ARO and FRO cell lines (data not shown).

### Forced expression of either HNF3*β*/FoxA2 or TTF-1 in thyroid carcinoma cells

**Figure 3:**
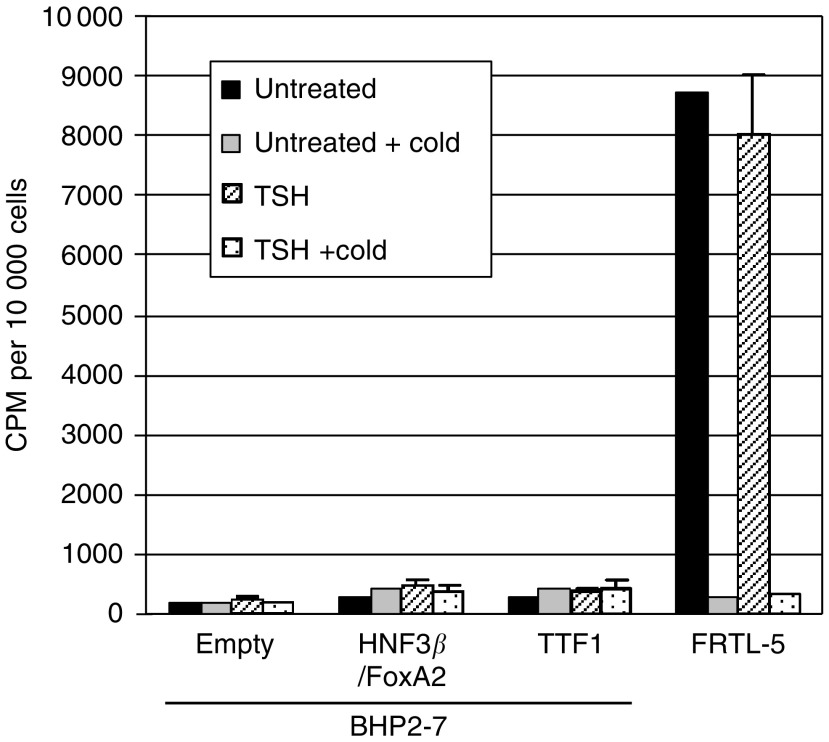
Forced expression of HNF3*β*/FoxA2 and TTF-1 in papillary thyroid carcinoma cells. Hepatocyte nuclear factor 3 *β*/FoxA2 and TTF-1 were overexpressed in BHP cells (subline 2–7). Uptake of ^125^I was measured in the transfected cells treated either with or without thyrotropin-stimulating hormone (TSH, 10 mU ml^−1^) for 24 h either in the presence or absence of cold sodium iodide. FRTL-5 cells, a normal rat thyroid cell line, were used as controls for the assay. Results represent the mean of three experiments of ^125^I counts per minute (CPM) per of either 10 000 cancer cells or normal cells.

Next, we transfected either the HNF3*β*/FoxA2 or the TTF-1 expression vector into papillary thyroid carcinoma cell lines. Neither HNF3*β*/FoxA2- nor TTF-1-expressing BHP cells (subline 2–7) had an increase in ^125^I uptake, when compared with normal FRTL-5 thyroid cells (Figure 3). Nevertheless, the forced expression of either HNF3*β*/FoxA2 or TTF-1 resulted in growth inhibition compared with cells transfected with an empty vector as measured by MTT assay (data not shown), suggesting that these transcription factors have antiproliferative activity in papillary thyroid carcinoma cells.

### Methylation status of *HNF3β/FoxA2* gene in papillary thyroid carcinoma cells

**Figure 4:**
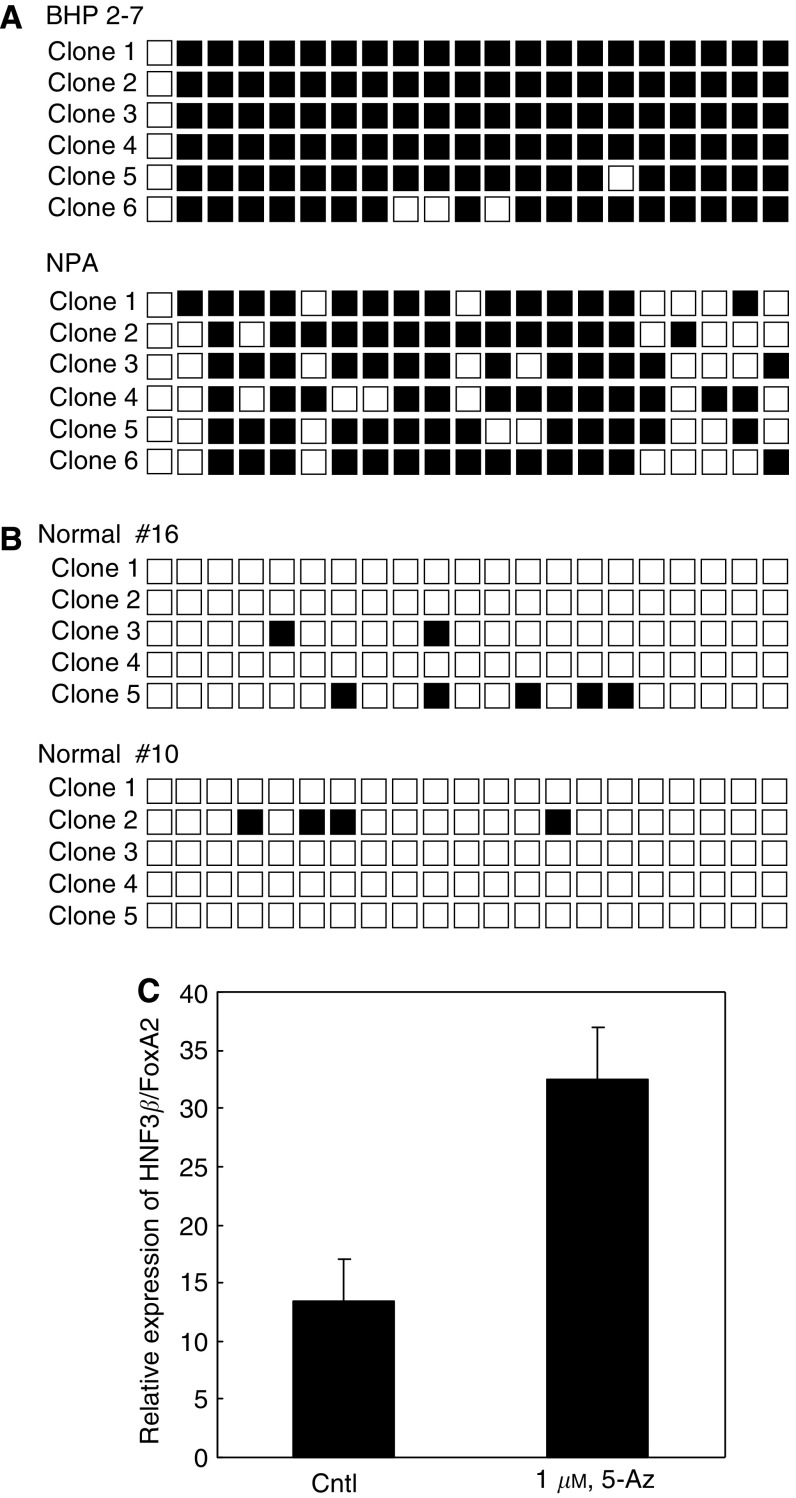
Methylation status of the CpG island of the HNF3*β*/FoxA2 gene. Genomic DNA of BHP (subline 2–7) and NPA cell lines (**A**), and two normal thyroid glands (**B**) were modified by sodium bisulphate. A total of 21 CpG sites in the CpG island of *HNF3β/FoxA2* gene were analysed by nucleotide sequencing. Methylated and unmethylated cytosines are shown by closed and open squares, respectively. (**C**) BHP cells (subline 2–7) were treated either with or without 1 *μ*M 5-Az, an inhibitor of DNA methyltransferase, for 96 h. Expression of HNF3*β*/FoxA2 mRNA was determined by quantitative RT–PCR. Levels of expression were determined as a ratio between target gene and the reference gene, *β*-actin. Cntl, control untreated cells. The level of HNF3*β*/FoxA2 expression is the mean of three samples.

The *HNF3β/FoxA2* gene has a CpG island in its promoter; and the region is often methylated in breast and lung cancers (Halmos *et al*, 2004; Miyamoto *et al*, 2005) prompting us to examine thyroid carcinoma cells. The great majority of the 21 CpG sites in the promoter were methylated in BHP (subline 2–7) and NPA papillary thyroid carcinoma cells (Figure 4A), as well as in the anaplastic thyroid carcinoma cell line FRO (data not shown). In contrast, the region was unmethylated in normal thyroid tissues (Figure 4B). Real-time PCR showed that the expression of HNF3*β*/FoxA2 mRNA was induced after the treatment of BHP cells (subline 2–7) with the demethylating agent 5-Az (1 *μ*M, 96 h) (Figure 4C). Taken together, these results suggest that the expression of the HNF3*β*/FoxA2 gene is epigenetically repressed in thyroid carcinoma cell lines.

### Forced expression of C/EBP*β* in thyroid carcinoma cells

**Figure 5:**
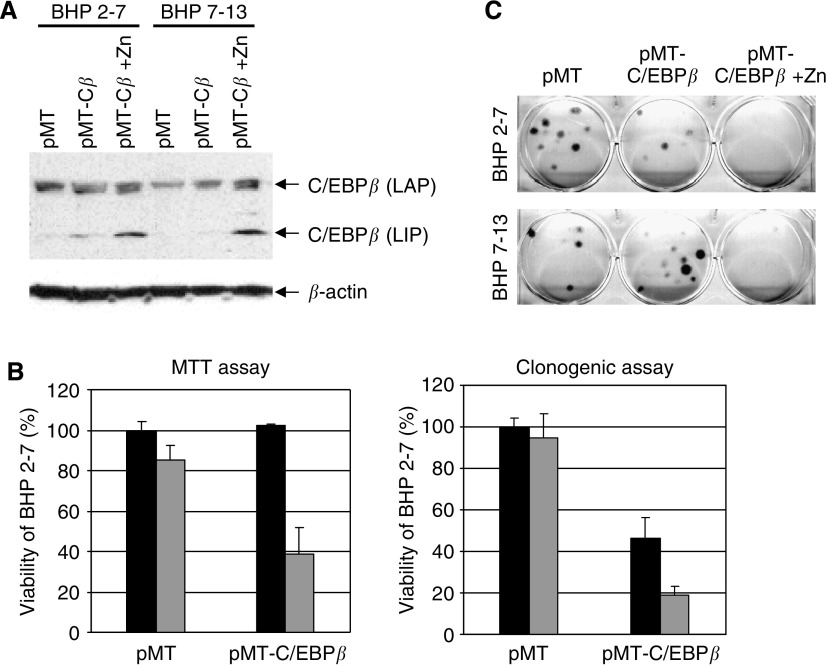
Forced expression of C/EBP*β* in papillary thyroid carcinoma cells. (**A**) Zinc-inducible expression vector, pMT-C/EBP*β* (pMT-C*β*), was stably transfected into BHP cells (sublines 2–7 and 7–13). These cells were cultured either with or without 1 *μ*M zinc. Expression of two isoforms of C/EBP*β*, LAP (liver-enriched transcriptional activating protein), and LIP (liver-enriched transcriptional inhibitory protein) in these cells was determined by Western blot analysis. (**B**) Viability of the transfected BHP cells (subline 2–7) was measured with MTT assay (left panel) and clonogenic soft agar assay (right panel) either in the presence or in the absence of zinc. Data represent the mean of three experiments. (**C**) Clonogenic growth of the Zn-inducible stably transfected BHP cells (sublines 2–7 and 7–13) grown in soft agar and stained with crystal violet. pMT cells were cultured with Zn as control.

Next, we placed a Zn-inducible C/EBP*β* expression vector into BHP cells (sublines 2–7 and 7–13) (Figure 5A). CCAAT/enhancer binding protein *β* has two isoforms, LAP (liver-enriched transcriptional activating protein) and LIP (liver-enriched transcriptional inhibitory protein). The smaller form of C/EBP*β* (LIP) clearly increased in the cells treated with zinc. Induction of C/EBP*β* expression resulted in a 60% growth reduction compared to the non-induced cells (Figure 5B, left panel). The more sensitive clonogenic soft agar assay showed that clonogenic growth decreased even in the absence of zinc, suggesting that this vector was ‘leaky’ resulting in C/EBP*β* expression even in the absence of zinc. Nevertheless, clonogenic growth decreased 50% in the presence of zinc compared with the absence of zinc (Figure 5B, right panel). Similarly, crystal violet staining demonstrated that C/EBP*β* had anti-growth activity in another subline, BHP 17-3 (Figure 5C). Taken together, these results suggested that forced expression of C/EBP*β* can cause growth inhibition in BHP papillary thyroid carcinoma cells.

### Cellular localisation of C/EBP*β* in human normal and papillary thyroid carcinoma tissues and cell lines

**Figure 6:**
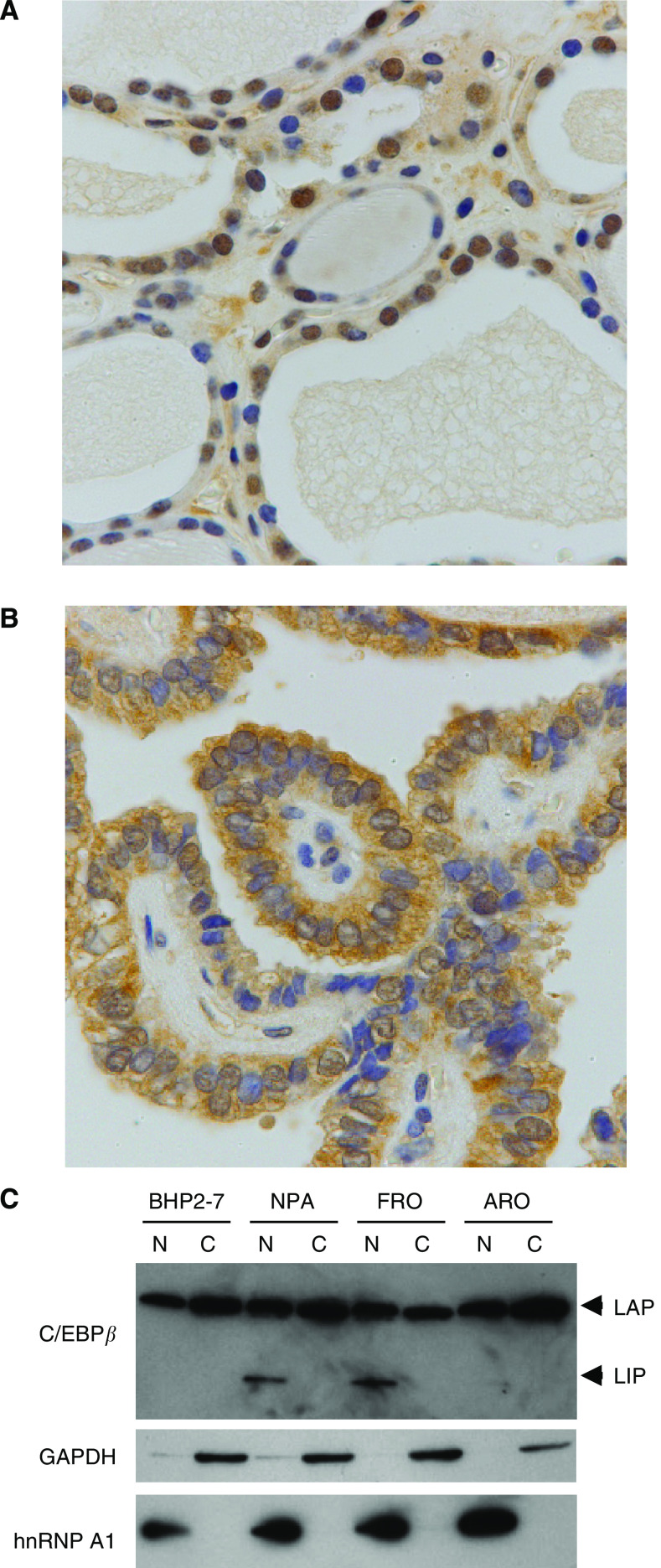
Cellular localisation of C/EBP*β* by immunohistochemistry in normal and papillary thyroid carcinoma tissues. Normal thyroid (**A**) and papillary thyroid carcinoma (**B**) tissues were immunohistochemically stained with anti-C/EBP*β* antibody. Photomicrographs are representative of three different samples of both normal thyroid and papillary thyroid carcinoma (data not shown). (**C**) Cellular fractionation of C/EBP*β* in papillary and anaplastic thyroid carcinoma cells. Papillary (BHP subline 2–7 and NPA) and anaplastic thyroid carcinoma (FRO and ARO) cells were fractionated into nuclear and cytoplasmic lysates, and the localisation of C/EBP*β* was determined by electrophoresis followed by Western blot analysis. Glyceraldehyde-3-phosphate dehydrogenase and hnRNP A1 are cytoplasm and nucleus markers, respectively. N, nuclear fraction; C, cytoplasmic fraction; LAP, liver-enriched transcriptional activating protein; LIP, liver-enriched transcriptional inhibitory protein.

To examine expression of C/EBP*β* in human thyroid tissues, normal and papillary thyroid carcinoma tissues were stained with anti-C/EBP*β* antibody. Immunohistochemistry revealed that C/EBP*β* signal was strongly detected in the nucleus in normal thyroid cells (Figure 6A). Interestingly, C/EBP*β* was detected in the cytoplasm and to a lesser extent the nucleus of papillary thyroid carcinoma cells (Figure 6B). The subcellular localisation of C/EBP*β* in four thyroid carcinoma cell lines (BHP2-7, NPA, FRO and ARO) was also determined by fractionation (Figure 6C). CCAAT/enhancer binding protein *β*-LAP was detected in both the nucleus and the cytoplasm. CCAAT/enhancer binding protein *β*-LIP, which has a dominant-negative activity against LAP, was expressed in NPA and FRO cell lines, and localised in the nucleus.

## Discussion

We attempted to induce differentiation and inhibit proliferation of thyroid carcinoma cells with various compounds and transcription factors; and in addition, we explored the abnormalities in endogenous expression of these transcription factors in thyroid carcinoma cells. Suberoylanilide hydroxamic acid modestly induced the expression of TPO, TG and TSHR; and the combination of SAHA and 1,25(OH)_2_D_3_ further enhanced the expression of NIS in BHP cell line. However, these agents were not potent stimulators of NIS expression level, when compared with expression levels found in normal thyroid tissues. Induced expression of these transcripts was about 10- to 100-fold lower than those found in normal thyroid cells. Therefore taken together, our data suggest that these compounds had little differentiation inducing activity and would be unlikely candidates to enhance the therapeutic value of radioactive iodine (^131^I) for the treatment of thyroid tumours. Notably, two histone deacetylase inhibitors, depsipeptide and Trichostatin A, have been shown to induce the expression of NIS and ^125^I uptake in several follicular and anaplastic thyroid carcinoma cell lines (Haugen, 2004).

Survey of the thyroid-specific genes showed that each promoter had transcription factor binding sites for HNF3*β*/FoxA2, TTF-1, C/EBP*β*, and Pax-8. Earlier studies using either the *TG* or *TPO* promoter found that they were activated by TTF-1 and HNF3*β*/FoxA2 in thyroid carcinoma cell lines (Sato and Di Lauro, 1996; Ros *et al*, 1999; Shimura *et al*, 2001). In addition, Pax-8 leads to the re-expression of NIS, TPO, TG, and TTF-1 mRNAs in ARO cells (Presta *et al*, 2005). Our present study demonstrated that forced expression of either HNF3*β*/FoxA2 or TTF-1 was unable to induce differentiation of the thyroid cancer cells as measured by NIS mRNA expression and radioiodine uptake. Similarly, co-transfection of HNF3*β*/FoxA2 and TTF-1 did not induce the expression of TPO, TG or TSHR mRNAs in BHP cells (data not shown), indicating that other molecule(s) might be required to induce endogenous mRNA expression of these thyroid-related differentiation genes. Interestingly, *HNF3β/FoxA2* is a methylated gene in breast and lung cancer cells; and overexpression of HNF3*β*/FoxA2 in a lung cancer cell line leads to growth arrest and apoptosis (Halmos *et al*, 2004; Miyamoto *et al*, 2005). Here, we report for the first time that the presence of aberrant methylation of *HNF3β/FoxA2* in thyroid carcinoma cell lines, and forced expression of the gene, resulted in growth inhibition.

Recently, Pomérance *et al* (2005) detected cytoplasmic localisation of C/EBP*β* in papillary thyroid carcinoma tissues. Our immunohistochemical analysis also showed cytoplasmic localisation of C/EBP*β* in papillary thyroid carcinoma tissues. In addition, we demonstrated by cell fractionation that C/EBP*β*-LAP was present in both the nucleus and the cytoplasm; in contrast, C/EBP*β*-LIP, a dominant-negative form of C/EBP*β*, was localised in the nucleus in NPA and FRO cells. Nucleocytoplasmic distribution of C/EBP*β* has been found in several other types of cancer. Human acute myeloid leukaemic cell line HL-60 showed cytoplasmic localisation of C/EBP*β*-LAP when Thr235 was phosphorylated, and the induction of differentiation and the inhibition of proliferation of these cells by 1,25(OH)_2_D_3_ resulted in nuclear translocation of the transcription factor (Marcinkowska *et al*, 2006). In other experiments, C/EBP*β* phosphorylation at Ser288 was associated with cytoplasmic localisation of the protein in human liver cancer cells; in contrast, normal liver cells had neither phosphorylation of Ser288 nor cytoplasmic C/EBP*β* (Buck *et al*, 2001). CCAAT/enhancer binding protein *β*-LAP and -LIP contain both nuclear localisation signal and nuclear export signal in their common C-terminal region (Williams *et al*, 1997). The N-terminal region, which is specific for C/EBP*β*-LAP, might contain a motif that causes cytoplasmic retention in thyroid cancer cells. In general, transcription factors including C/EBP*β* function in the nucleus, suggesting that deregulation of nuclear localisation of C/EBP*β* leads to functional deficiency and result in cell abnormalities.

In summary, we found that the thyroid cancer cells had decreased the expression of TTF-1 and HNF3*β*/FoxA2; and their forced re-expression was associated with decreased cell growth. In addition, methylation of *HNF3β/FoxA2* and inappropriate cellular localisation of C/EBP*β* were identified as novel abnormalities. Future studies will screen for small molecules that can induce expression of these transcription factors resulting in a unique therapy for thyroid cancer.

## Figures and Tables

**Table 1:** Primer and probe sequences used for real-time RT–PCR

**Gene name**		**Sequence**	**Melt. Temp.**	**Size**
NIS	S	CCCTCATCCTGAACCAAGTG	—	230 bp
	AS	GATCCGGGAGTGGTTCTG		
	probe	CTGGACATCTGGGCGTCGCTC		
				
TPO	S	ACCTCGACGGTGATTTGCA	82.0°C	71 bp
	AS	CCGCCTGTCTCCGAGATG		
				
TG	S	GTGCCAACGGCAGTGAAGT	81.0°C	87 bp
	AS	TCTGCTGTTTCTGTAGCTGACAAA		
				
TSHR	S	CCCAGCTTACCGCCCAGT	81.0°C	79 bp
	AS	TAGAAAATGCATGACTTGGAATAGTTC		
				
HNF3β/FoxA2	S	AAGACCTACAGGCGCAGCTA	87.0°C	214 bp
	AS	CCTTCAGGAAACAGTCGTTGA		
				
TTF-1	S	GCCGTACCAGGACACCATGAG	86.0°C	265 bp
	AS	CAGGTACTTCTGTTGCTTGAAG		
				
Pax-8	S	AAGTCCAGCATTGCGGCACA	84.0°C	331 bp
	AS	GAGGGAAGTGCTTATGGTCC		
				
C/EBP*α*	S	TGGACAAGAACAGCAACGAG	—	130 bp
	AS	TTGTCACTGGTCAGCTCCAG		
	probe	CACCTTCTGCTGCGTCTCCACGTT		
				
C/EBP*β*	S	GACAAGCACAGCGACGAGTA	—	102 bp
	AS	GTGCTGCGTCTCCAGGTT		
	probe	ATC TTG GCC TTG TCG CGG CTC TT		
				
18S	S	AAACGGCTACCACATCCAAG	83.0°C	155 bp
	AS	CCTCCAATGGATCCTCGTTA		
				
*β*-actin	S	CCCAGATCATGTTTGAGACC	87.0°C	154 bp
	AS	AGGGCATACCCCTCGTAGAT		

AS, anti-sense primer; Melt. Temp., melting temperature; RT–PCR, reverse transcription-polymerase chain reaction; S, sense primer.
